# Association between preferred language and use of mental health services among home care recipients with schizophrenia spectrum and other psychotic disorders: A retrospective cohort study in Ontario, Canada, 2010 to 2015

**DOI:** 10.1371/journal.pmen.0000013

**Published:** 2024-07-22

**Authors:** Sarah Carson, Mary M. Scott, Emily Rhodes, Ricardo Batista, Patrick Tang, Denis Prud’homme, Peter Tanuseputro, Colleen Webber

**Affiliations:** 1 Faculty of Medicine, University of Ottawa, Ottawa, Ontario, Canada; 2 Ottawa Hospital Research Institute, Ottawa, Ontario, Canada; 3 ICES, Ontario, Canada; 4 Bruyère Research Institute, Ottawa, Ontario, Canada; 5 Institut du Savoir Montfort, Ottawa, Ontario, Canada; 6 Division of Palliative Care, Department of Medicine, University of Ottawa, Ottawa, Ontario, Canada; University of Milano–Bicocca: Universita degli Studi di Milano-Bicocca, ITALY

## Abstract

Language is an important demographic factor that may impact patients’ interactions with the healthcare system. This may become more apparent for individuals with a mental illness. The objective of this study was to evaluate whether individual language was associated with the use of inpatient and outpatient psychiatric services among home care recipients with schizophrenia spectrum and other psychotic disorders. We conducted a population-based retrospective cohort study using health administrative data. The study population included all individuals aged 18–105 with schizophrenia spectrum and other psychotic disorders, identified via a validated algorithm, who were receiving home care in Ontario, Canada between January 1, 2010 and December 31, 2015. Home care data was used to identify patients’ primary language, categorized as Anglophone, Francophone, or Allophone. Mental health service use was evaluated over a one-year period following their first home care assessment in the study period and included the rate of outpatient psychiatrist visits and mental health-related emergency department (ED) visits and hospitalizations. Multivariable regression evaluated the association between primary language and mental health service use. The cohort consisted of 9,436 patients (85.8% Anglophones, 3.3% Francophones, 11.0% Allophones). Mental health service use was low among all linguistic groups during the one-year study period, with 53.4% with no outpatient psychiatrist visits and 83.3% and 83.0% with no mental health-related hospitalizations or ED visits, respectively. Francophones had a higher rate of mental health-related hospitalizations compared to Anglophones (adjusted relative risk = 1.36, 95% confidence interval 1.02–1.80), with no differences in hospitalization rates between Allophones and Anglophones. Language was not associated with the rate of outpatient psychiatrist visits or mental health-related ED visits. Mental health service use among homecare patients with schizophrenia spectrum and other psychotic disorders was low. While Francophones had a higher hospitalization rate than Anglophones, language was otherwise not associated with mental health service use.

## Introduction

Language is an important demographic factor that can impact both healthcare use and patients’ experiences in the healthcare system. Language barriers between patients and their healthcare providers have been shown to negatively impact access to and quality of healthcare, in addition to patient safety [1–6]. In a Canadian context, there exists considerable linguistic diversity, which has important implications for patients and their interaction with the healthcare system. At a national level, English and French are recognized as the official languages of Canada [7], while in Ontario, the setting of this study, the official language is English. As reported in 2021, 77.4% of the Ontario population speak primarily English at home, 1.8% speak French, and 15.7% speak a language other than English or French (i.e., Allophones). This is similar to reported mother tongue, with 65.1%, 3.3%, and 26.9% of Ontarians reporting their mother tongue is English, French, or another language [8]. While some healthcare facilities in Ontario are designated as offering French-language services through the French Language Services Act, there still remains challenges with language discordance between patients and providers. The majority of physicians in Ontario are Anglophone, meaning that compared to Anglophone patients, patients who are members of linguistic minorities (i.e., Francophones and Allophones) are less likely to be cared for by a physician who speaks their language [9]. This provides important context for how linguistic heterogeneity may influence a patient’s experience with the healthcare system in Canada.

Recent work has shown that among home care recipients admitted to hospital, receipt of care from physicians who do not speak the patients’ primary language significantly increased the risk of adverse events and in-hospital deaths and the length of hospital stays [9]. Home care recipients are typically older adults with frailty who need additional supports, such as nursing and personal care, to live safely in the community. Home care services include short- and long-term supports, as well as specialized programs such as end-of-life care [10]. For home care recipients with mental illness, linguistic barriers with care providers may be particularly challenging given that mental healthcare often relies on verbal communication for assessment, diagnosis, and management [11]. To date, few studies have examined the relationship between language and mental health service use among individuals with mental illness [12–15].

Individuals with schizophrenia spectrum and other psychotic disorders, including schizophrenia, schizoaffective disorder and psychosis not otherwise specified, have high healthcare needs and are disproportionately high users of the healthcare system [16]. These diagnoses are associated with poor psychiatric and medical outcomes, such as psychiatric and physical comorbidities, and high rates of hospitalizations and mortality [17–25]. There is a well-established physical healthcare gap for individuals with schizophrenia and other psychotic disorders. Despite an increased risk of physical comorbidities, individuals often face challenges accessing care for their physical health needs and are at risk of poor-quality care and clinical management [26]. Individuals with schizophrenia spectrum and other psychotic disorders have a decreased lifespan compared to the general population, congruent with the observed two to three-fold increase in mortality rates for people with these diagnoses [23, 27–29]. Previous studies have found that lack of access to quality health care can, in part, explain these poorer health outcomes and higher mortality rates [30, 31].

The objective of this study was to evaluate the relationship between primary language and the use of mental health services by individuals with schizophrenia spectrum and other psychotic disorders receiving home care in Ontario, Canada. Primary language was classified as Anglophone, Francophone, or Allophone (i.e., a language other than English or French). Mental health services included outpatient psychiatric care and mental health-related hospitalizations and emergency department (ED) visits. Understanding the relationships between language and the utilization of mental health services for individuals with schizophrenia spectrum and other psychotic disorders is important to identify and address any potential barriers to care for individuals with these mental illnesses.

## Methods

### Ethics statement

ICES is a prescribed entity under section 45 of Ontario’s Personal Health Information Protection Act. Section 45 authorizes ICES to collect personal health information, without consent, for the purpose of analysis or compiling statistical information with respect to the management of, evaluation or monitoring of, the allocation of resources to or planning for all or part of the health system. Projects conducted under section 45, by definition, do not require review by a Research Ethics Board. This project was conducted under section 45 and approved by ICES’ Privacy and Compliance Office.

### Study design and data sources

We conducted a population-based retrospective cohort study of all home care recipients with schizophrenia spectrum and other psychotic disorders in Ontario, Canada between January 1, 2010 and December 31, 2015. Ontario has a population of over 14 million residents (based on the 2021 Census) and a single payer, publicly funded healthcare system [32]. The Ontario Health Insurance Program (OHIP) covers the costs of physician and hospital services for all residents, as well as home care for residents deemed eligible based on assessed need [33]. We used linked administrative health databases held at ICES (previously known as the Institute for Clinical Health Evaluative Sciences) to conduct this study [34, 35]. ICES is an independent, non-profit research institute in Ontario whose legal status under Ontario’s health information privacy law allows it to collect and analyze health care and demographic data, without consent, for health system evaluation and improvement. Specific data sources used in this study include: (1) home care assessment data which is collected on a routine basis at home care initiation and every six months on all long-stay (i.e., expected to receive services for >60 days) home care recipients via the Resident Assessment Instrument–Home Care (RAI-HC), a standardized assessment tool developed by InterRAI [36]; (2) physician billing claims via OHIP, which capture the service provided and main diagnosis associated with all physician encounters; (3) hospital discharge records via the Canadian Institute for Health Information Discharge Abstract Database (CIHI-DAD) and the Ontario Mental Health Reporting System (OMHRS); (4) ED visit records via the National Ambulatory Care Reporting System (NACRS); (5) prescription drug dispensing data via the Ontario Drug Benefit Program, (6) demographic data via the Registered Persons Database (RPDB), which contains sex, birth and death dates, and yearly postal codes for all individuals eligible for OHIP, and (7) 2016 Canadian Census data. Details of these data sources are provided in S1 Table. These datasets were linked using unique encoded identifiers and analyzed at ICES.

### Study population

The study population included all individuals who received long-stay home care services in Ontario between April 1, 2010 and March 31, 2015 and who had a pre-existing diagnosis of schizophrenia spectrum and other psychotic disorders. Study follow-up began at individuals’ first (index) RAI-HC assessment within the study period. We chose to study long-stay home care recipients as they have information on their preferred language captured via their RAI-HC assessment. The diagnosis of schizophrenia spectrum and other psychotic disorders was ascertained via an algorithm based on at least 1+ prior inpatient hospitalization with relevant diagnosis codes (International Classification of Disease (ICD)-9 codes 293.81, 293.82, 295, 297, 298, ICD-10 codes F06.0, F06.1, F06.2, F20, F22-F29, Diagnostic and Statistical Manual of Mental Disorders 4^th^ edition (DSM-IV): 295, 297, 298). Individuals who met this algorithm criteria based on their healthcare utilization at any point prior to their index RAI-HC assessment were included. This definition is used by the ICES Mental Health and Addictions Program reporting to identify hospitalizations for schizophrenia spectrum and other psychotic disorders [37]. We excluded individuals who were under age 18 or over age 105 at the start of follow-up. We excluded individuals under the age of 18 for two reasons. First, given the average age of onset of schizophrenia spectrum disorders, we expected that diagnoses in those under the age of 18 may reflect misdiagnoses. Further, mental health-related acute care in individuals under the age of 18 is not reliably captured in the administrative data. The exclusion of individuals age >105 was used as it is expected that these age calculations may be errors due to data quality issues in the administrative data. We also excluded individuals with an invalid death date, who were non-Ontario residents, or who were not eligible for OHIP coverage during follow-up.

### Study variables

#### Exposure

Home care recipients’ language was obtained from the index RAI-HC assessment [36]. RAI-HC assessments identify the individual’s primary language, defined as a person’s preferred language for day-to-day communication. This is determined by trained assessors via recipient and family interview, observation, and documentation on the home care referral. We identified three main linguistic groups: Anglophone, Francophone, and Allophone (i.e., the preferred language was one other than English or French). Assessors can only identify one preferred language, and the data do not identify if individuals speak language(s) other than their preferred language.

#### Outcomes

We measured the use of three types of mental health services in the one-year follow-up period as our primary outcomes: outpatient psychiatric services, mental health-related hospitalizations, and mental health-related ED visits. We used OHIP physician billing claims to identify all outpatient visits to a psychiatrist, counting a maximum of one visit per day. Mental health-related hospitalizations were identified as any discharge record in OMHRS, or any hospitalization in CIHI-DAD with any diagnosis of mental and behavioural disorders (ICD-10 F06-F99) registered as the primary diagnosis or any secondary diagnosis of intentional self-harm, poisoning or contact with sharp object events of undetermined intent (ICD-10: X60-X84, Y10-Y19, Y28) when primary diagnosis was not a mental or behavioural condition. We used NACRS to identify emergency department visits with any diagnosis of mental and behavioural disorders registered as primary diagnosis (ICD-10: F00-F99) or any secondary diagnoses of intentional self-harm, poisoning or contact with sharp object events of undetermined intent (ICD-10: X60-X84, Y10-Y19, Y28) when primary diagnosis is not a mental and behavioural condition.

#### Covariates

Baseline sociodemographic variables obtained from the RPDB included age at index, sex, and postal code to assign area-level income quintile and rurality (urban vs. rural) based on 2016 Canadian Census data. Comorbid conditions were defined using previously developed algorithms which use diagnosis codes on physician billing claims and hospitalization records, and drug dispensing data. They included acute myocardial infarction, arrhythmia, asthma, cancer, congestive heart failure, chronic obstructive pulmonary disease (COPD), coronary artery disease, dementia, diabetes, hypertension, inflammatory bowel disease, osteoarthritis, osteoporosis, renal disease, rheumatoid arthritis, and stroke. Each of these conditions was ascertained based on healthcare encounters in the two years prior to the start of study follow-up. We used a count of these conditions, as a measure of comorbid disease burden [38–47]. RAI-HC data was used to determine if individuals had an informal helper who lived with them (yes, no, or no informal helper), as well as to measure individuals’ independence in activities of daily living (ADLs) via the ADL Self-Performance Hierarchy (scored from 0-independent in ADLs to 6-total dependence), cognition via the Cognitive Performance Scale (CPS) (scored 0-cognition intact to 6-severely impaired cognition), health instability via the Changes in Health, End-Stage Disease, Signs and Symptoms (CHESS) scale (scored 0-no health instability to 5-very high health instability) [48]. These covariates were selected a priori as potential confounders of the relationship between language and the use of mental health service use. All covariates were included as confounding variables in the adjusted regression models. Detailed descriptions of all study variables are provided in S2 and S3 Tables.

### Statistical analysis

We used descriptive statistics (mean and standard deviation, median, counts, and proportions) to describe and compare home care recipient characteristics across linguistic groups. We reported the distribution (mean, standard deviation, median, and count as 0, 1–2, or 3+ encounters) of outpatient psychiatric visits, hospitalizations, and ED visits across linguistic groups. We compared the distribution of these outcomes across linguistic groups using bivariate statistical tests (chi-square tests, analysis of variance). We then evaluated the association between language and the count of each outcome using multivariable negative binomial regression models. This regression model was chosen as it fits count data, such as the number of healthcare encounters. Anglophones were the reference group in all regression models. The models estimated the adjusted rate ratio (RR) and 95% confidence interval (CI) for each outcome, comparing Francophones vs. Anglophones, and Allophones vs. Anglophones. All models were adjusted for age (modelled continuously via 3-knot restricted cubic spline), sex (reference = female), urban/rural status (reference = urban), area-level income quintile (reference = quintile 5, highest income quintile), number of comorbid conditions (modelled continuously via 3-knot restricted cubic spline), presence of a caregiver in the home (reference = yes), ADL independence (reference = 0), cognition via CPS (reference = 0 vs. 1, 2, 3, or 4+), and health instability via CHESS score (reference = 0 vs. 1, 2, or 3+) [49]. All statistical tests were two-tailed, and the significance threshold was set at 0.05. SAS 9.4 (SAS Institute, Cary, NC) was used for all analyses.

## Results

In total, 9,436 home care recipients with a schizophrenia spectrum and other psychotic disorders diagnosis met eligibility criteria and were included (see S1 Fig for study flow diagram). Most were Anglophones (85.7%), while Francophones and Allophones represented 3.3% and 11.0% of the cohort, respectively. The baseline characteristics of the home care recipients are reported in Table 1. Allophones were slightly older (mean 69.2 years) than Anglophones (mean 63.5 years) and Francophones (mean 65.8 years). Roughly one-third of each linguistic group was male. Francophones had the highest proportion of individuals living in rural areas (25.6%). Allophones were the most likely to live with an informal caregiver (51.6%). Roughly one-third of each linguistic group lived in areas that were in the lowest income quintile, with no differences across the groups.

**Table 1:** Baseline sociodemographic characteristics of 9,436 home care recipients with schizophrenia spectrum and other psychotic disorders (2010–2015), by linguistic group.

	Anglophone N = 8087	Francophone N = 313	Allophone N = 1036	p-value
**Age, mean (SD) [median]**	63.5 (14.2) [64]	65.8 (13.4) [67]	69.2 (14.4) [72]	<0.001
**Sex–n (%)**				0.15
A. Female	5109 (63.2)	213 (68.1)	642 (62.0)	
B. Male	2978 (36.8)	100 (31.9)	394 (38.0)	
**Income quintile–n (%)**				0.01
A. 1 (lowest)	3000 (37.1)	105 (33.5)	363 (35.0)	
B. 2	1626 (20.1)	75 (24.0)	229 (22.1)	
C. 3	1300 (16.1)	50 (16.0)	164 (15.8)	
D. 4	1137 (14.1)	50 (16.0)	143 (13.8)	
E. 5 (highest)	929 (11.5)	32 (10.2)	110 (10.6)	
F. Missing	95 (1.2)	1 (0.3)	27 (2.6)	
**Residential status–n (%)**				<0.001
A. Urban	7169 (88.6)	232 (74.1)	1009 (97.4)	
B. Rural	904 (11.2)	81 (25.9)	26 (2.5)	
C. Missing	14 (0.2)	0 (0.0)	1 (0.1)	
**Informal caregiver lives with patient–n (%)**				<0.001
A. Yes	2770 (34.3)	98 (31.3)	535 (51.6)	
B. No	4133 (51.1)	171 (54.6)	414 (40.0)	
C. No informal caregiver	1184 (14.6)	44 (14.1)	87 (8.4)	

SD: standard deviation

The health and functional characteristics of the cohort are reported in Table 2. Francophones had a higher number of comorbid conditions compared to Anglophones and Allophones. Across all linguistic groups, more than half had a comorbid non-psychotic mood or anxiety disorder (52.0%, 55.0%, and 52.7% for Anglophone, Francophone, and Allophone, respectively). The proportion of home care recipients with cancer and respiratory conditions was higher among Anglophones (Cancer: 15.0%, Asthma: 21.1%, COPD: 22.2%) and Francophones (Cancer: 12.8%, Asthma: 25.2%, COPD: 27.2%). Allophones were more likely to have dementia (34.7%). Compared to Anglophones (39.0%), the proportion of home care recipients with diabetes was higher among Francophones (46.6%) and Allophones (46.9%). While Anglophones and Francophones had similar cognitive performance and independence in ADLs, Allophones had a higher degree of cognitive impairment and greater dependence in ADLs. Compared to Anglophones and Allophones, Francophones had a higher proportion of health instability, based on their CHESS score.

**Table 2:** Health and functional status of 9,436 home care recipients schizophrenia spectrum and other psychotic disorders (2010–2015), by linguistic group.

	Anglophone N = 8087	Francophone N = 313	Allophone N = 1036	p-value
**Number of comorbid conditions, mean (SD) [median]**	4.2 (2.1) [4]	4.6 (2.1) [4]	4.4 (2.1) [4]	0.003
**Comorbid conditions–n (%)**				
A. Asthma	1708 (21.1)	79 (25.2)	146 (14.1)	<0.001
B. Cancer	1210 (15.0)	40 (12.8)	113 (10.9)	0.002
C. Congestive heart failure	1046 (12.9)	43 (13.7)	168 (16.2)	0.01
D. COPD	1793 (22.2)	85 (27.2)	105 (10.1)	<0.001
E. Coronary artery disease	1535 (19.0)	70 (22.4)	232 (22.4)	0.01
F. Dementia	2035 (25.2)	75 (24.0)	360 (34.7)	<0.001
G. Diabetes	3153 (39.0)	146 (46.6)	486 (46.9)	<0.001
H. Non-psychotic mood and anxiety disorder	4204 (52.0)	172 (55.0)	546 (52.7)	0.55
I. Stroke	703 (8.7)	32 (10.2)	124 (12.0)	0.002
**Cognition via CPS–n (%)**				<0.001
A. Intact	1858 (23.0%)	61 (19.5%)	146 (14.1%)	
B. Borderline intact	1745 (21.6%)	67 (21.4%)	211 (20.4%)	
C. Mild impairment	2588 (32.0%)	118 (37.7%)	348 (33.6%)	
D. Moderate impairment	1398 (17.3%)	49 (15.7%)	200 (19.3%)	
E. Moderate severe impairment	155 (1.9%)	≤5*	45 (4.3%)	
F. Severe impairment	301 (3.7%)	8–16*	75 (7.2%)	
G. Very severe impairment	42 (0.5%)	≤5*	11 (1.1%)	
**ADL Self-Performance–n (%)**				<0.001
A. Independent	4583 (56.7)	174 (55.6)	502 (48.5)	
B. Supervision required	1158 (14.3)	53 (16.9)	155 (15.0)	
C. Limited impairment	1196 (14.8)	57 (18.2)	175 (16.9)	
E. Extensive assistance required (1)	620 (7.7)	14 (4.5)	105 (10.1)	
E. Extensive assistance required (2)	266 (3.3)	10–14*	52 (5.0)	
F. Dependent	214 (2.6)	≤5*	35 (3.4)	
G. Total dependence	60 (0.6)	0 (0.0)	12 (1.2)	
**CHESS Scale–n (%)**				0.04
A. No health Instability	2680 (33.1)	96 (30.6)	371 (35.8)	
B. Minimal health instability	2521 (31.2)	87 (27.8)	320 (30.9)	
C. Low health instability	1871 (23.1)	72 (23.0)	233 (22.5)	
D. Moderate health instability	814 (10.1)	42 (13.4)	92 (8.9)	
E. High health instability	192 (2.4)	11–15*	15–19*	
F. Very high health instability	9 (0.1)	≤5*	≤5*	

*Results have been suppressed to ensure no reporting of small cells

SD: standard deviation; COPD: chronic obstructive pulmonary disease; CPS: Cognitive Performance Scale; ADL: activities of daily living; CHESS: Changes in Health, End-Stage Disease, Signs and Symptoms.

### Mental health service utilization

The use of mental health services is presented in Table 3. Over the one-year follow-up, over half of the cohort (53.4%) had no outpatient psychiatric visits, while 28.8% had three or more visits (mean 2.5). Francophones had the highest proportion of individuals with no outpatient psychiatric visits (59.1%), while Allophones had the highest proportion of individuals with three or more outpatient psychiatric visits (31.5%, p = 0.01). Mental health-related hospitalizations were uncommon, with 83.3% with no mental health-related hospitalizations (mean 0.2). Counts of mental health-related hospitalizations did not differ across the linguistic groups (p = 0.12). Mental health-related ED visits were also uncommon, with 83.3% with zero visits (mean 0.4). Allophones had the highest proportion of individuals with no mental health-related ED visits (87.2%), while Francophones had the highest proportion of individuals with three or more mental health-related ED visits (p<0.001).

**Table 3:** Mental health service use among 9,436 home care recipients schizophrenia spectrum and other psychotic disorders (2010–2015), by linguistic group.

	Overall N = 9436	Anglophone N = 8087	Francophone N = 313	Allophone N = 1036	P-value
**Outpatient psychiatric visits in 1 year, mean (SD) [median]**	2.5 (5.7) [0]	2.6 (5.8) [0]	2.2 (6.9) [0]	2.4 (4.3) [1]	0.50
**Visit count–n (%)**					
A. 0	5041 (53.4)	4348 (53.8)	185 (59.1)	508 (49.0)	0.01
B. 1–2	1679 (17.8)	1424 (17.6)	53 (16.9)	202 (19.5)	
C. 3+	2716 (28.8)	2315 (28.6)	75 (24.0)	326 (31.5)	
**Mental health-related hospitalizations, mean (SD) [median]**	0.2 (0.7) [0]	0.2 (0.7) [0]	0.3 (0.8) [0]	0.2 (0.7) [0]	0.08
**Hospitalization count–n (%)**					
A. 0	7864 (83.3)	6733 (83.3)	248 (79.2)	883 (85.2)	0.12
B. 1–2	1408 (14.9)	1206 (14.9)	59 (18.8)	140 (13.5)	
C. 3+	164 (1.7)	145 (1.8)	6 (1.9)	13 (1.3)	
**Mental health-related ED visits, mean (SD) [median]**	0.4 (2.1) [0]	0.4 (2.2) [0]	0.5 (1.5) [0]	0.2 (1.1) [0]	0.03
**ED visit count–n (%)**					
A. 0	7862 (83.3%)	6712 (83.0)	247 (78.9)	903 (87.2)	<0.001
B. 1–2	1230 (13.0)	1062 (13.1)	52 (16.6)	116 (11.2)	
C. 3+	344 (3.6)	313 (3.9)	14 (4.5)	17 (1.6)	

SD: Standard deviation, ED: emergency department

The adjusted rate ratios (RR) and 95% CIs of mental health service utilization rates across the linguistic groups are presented in Table 4. There was a slightly higher adjusted rate of mental health-related hospitalizations among Francophones compared to Anglophones (RR 1.36, 95% CI 1.02–1.80). There was no difference in the rate of mental health-related hospitalizations between Allophones and Anglophones (RR 1.01, 95% CI 0.84–1.22). There was no difference in the adjusted rates of outpatient psychiatric visits or mental health-related ED visits for Francophones or Allophones when compared to Anglophones.

**Table 4:** Adjusted* regression results evaluating differences in mental health service use among 9,436 home care recipients schizophrenia spectrum and other psychotic disorders (2010–2015), by linguistic group.

	Outpatient psychiatric visits RR (95% CI)	Mental health-related hospitalizations RR (95% CI)	Mental health-related ED visits RR (95% CI)
**Language**			
Anglophone	1.00 (ref)	1.00 (ref)	1.00 (ref)
Francophone	1.03 (0.82–1.28)	1.36 (1.02–1.80)	1.21 (0.87–1.70)
Allophone	1.09 (0.96–1.24)	1.01 (0.84–1.22)	0.96 (0.77–1.20)

*Adjusted for age, sex, income quintile, rurality, number of comorbid conditions, cognition via Cognitive Performance Scale, function via the Activities of Daily Living Self-Performance Hierarchy, presence of an informal caregiver in the home, and health instability via the Changes in Health, End-Stage Disease, Signs and Symptoms Scale.

RR: relative risk; CI: confidence interval; ED: emergency department

## Discussion

In this study, we explored the associations between individual language and the rate of outpatient psychiatric visits in addition to mental health related hospitalizations and ED visits among community-based home care recipients with schizophrenia spectrum and other psychotic disorders. While we observed that Francophone individuals had a higher rate of mental health-related hospitalizations than Anglophone individuals, rates of hospitalizations did not differ between Allophones and Anglophones. Further, there were no differences in rates of outpatient psychiatric visits or mental health-related ED visits between linguistic groups. Overall, mental health service use observed in this study was low, regardless of linguistic group, with more than half of the cohort having no outpatient psychiatric visits and almost 80% or more having no mental health-related hospital admissions or ED visits.

Our findings contrast those of previous studies that have reported that language is associated with meaningful differences in mental healthcare use and healthcare quality for individuals with a mental illness. A study of individuals admitted to psychiatric facilities in Ontario reported that Francophone and Allophone patients were less likely have daily contact with a psychiatrist in the three days of admission compared to Anglophone patients [50]. In another study of patients enrolled in mental health case management programs in Toronto, Canada, individuals with limited English proficiency, defined as having a preferred language other than English, were more likely to report unmet health needs, including needs related to their physical health, psychological distress, and psychotic symptoms [51]. Limited English proficiency has also been associated with reduced mental health service use among Latino immigrant adults [13]. Qualitative studies have identified language as a barrier to high quality mental health service, with patients reporting that language discordance between patients and providers has a negative impact on the therapeutic relationship, communication, and quality of care [52, 53]. There are several potential factors that may be contributing to our contradictory findings. First, many previous studies included individuals with a range of mental illnesses, while this study focused on schizophrenia spectrum and other psychotic disorders. The severity of symptoms and higher level of healthcare need among individuals with schizophrenia spectrum and other psychotic disorders compared to other mental illnesses may lessen the impact of language barriers on care. Second, we studied home care recipients, which was necessary to measure patient language. As a result, our study population was older than the general population of individuals with schizophrenia spectrum and other psychotic disorders and had a higher burden of comorbid conditions [54]. With diagnoses of schizophrenia spectrum and other psychotic disorders typically occurring in early adulthood, the individuals in our study have likely lived with their condition for many years and may have established networks of care providers who can provide language-concordant care. Further, the availability of home care services, which often include nursing, may offset the need for psychiatric care and may help connect individuals with other community-based services that reduce their need for psychiatric care. However, it is not clear whether home care services can actually support the complex mental health needs of those with schizophrenia spectrum and other psychotic disorders [55]. Finally, home care recipients with schizophrenia spectrum and other psychotic disorders, regardless of language spoken, may also have more informal supports, such as family caregivers, than individuals with schizophrenia spectrum and other psychotic disorders who are not receiving home care. These informal supports may minimize any effects of language barriers, for instance, through managing appointments and offering interpreting services.

This study had several strengths. The population-based data used in this study provided a large sample size with sufficient power to examine differences in linguistic groups. The healthcare utilization data captured all healthcare encounters with psychiatrists in Ontario, including in inpatient and outpatient settings. The homecare assessment data captured rich detail on individuals’ health, function, cognition, and caregiver support, which may be important confounders of the relationship between language and psychiatric care. This study had several limitations. First, the diagnosis of schizophrenia spectrum and other psychotic disorders in this study was ascertained via a non-validated algorithm based on at least 1+ prior inpatient hospitalization with a relevant diagnosis code. A previous study developed and validated an algorithm for chronic psychotic illnesses based on 1+ hospitalization for schizophrenia, schizoaffective disorder, or psychosis not otherwise specified, with a sensitivity of 79.4% and specificity of 80.7% [56]. Our study included more diagnosis codes as a part of the case definition, which may increase sensitivity at the cost of specificity. The algorithm in this study is also based on only prior hospitalizations which may exclude less severe cases of psychotic disorder that were only seen in outpatient settings. Second, primary language was determined based on the RAI-HC home care assessment, where interviewers determine the home care recipient’s primary language through observation and, if needed, by asking the recipient to indicate their primary language [36]. The RAI-HC assessments only capture one preferred language, and it is uncertain how interviewers determined the primary languages of multilingual patients. As a result, individuals may have been incorrectly classified into a linguistic group. Additionally, language proficiency is not assessed so it is possible that a home care recipient is identified as Francophone or Allophone while having high English proficiency. However, the primary language registered in the RAI-HC database generally concurs (kappa = 0.76) with self-reported language from the Canadian Community Health Survey, reflecting the language regularly spoken at home [57]. Third, our study only examined outpatient mental healthcare provided by psychiatrists. While psychiatrists often treat the most complex mental health cases, the role of primary care in the treatment of mental health was not assessed in this study. However, while family physicians offer the bulk of outpatient mental healthcare in Canada, many do not feel comfortable managing serious mental health disorders such as schizophrenia [58]. Therefore, it is likely that many patients with schizophrenia require care by a psychiatrist to support their mental health needs. Fourth, we studied a home care population, resulting in a study cohort with an average age of approximately 64 years. Patients with schizophrenia spectrum and other psychotic disorders tend to have higher rates of healthcare utilization at a younger age, with use decreasing as they get older due to stabilization in the illness [59, 60]. It is possible that our findings do not generalize to younger individuals with schizophrenia spectrum and other psychotic disorders who have only recently been diagnosed. Fifth, there may be uncontrolled confounding in our results due to our reliance on health administrative data that does not capture important information, such as disease severity and other clinical indications for care, healthcare preferences and beliefs, and many community-based mental health supports (e.g., counsellors). Finally, our study examined healthcare utilization between 2010 and 2015. It is possible that some of our findings do not reflect current rates of mental health service use. However, we think that the relationships between language and the use of mental health services are likely generalizable and relevant to current practice given the body of evidence linking language to disparities in healthcare use and health outcomes [1, 3, 5, 9]. However, further research using more contemporaneous data is needed.

## Conclusions

The rate of outpatient psychiatric care and mental health-related ED visits among individuals with schizophrenia spectrum and other psychotic disorders did not differ between linguistic groups. While Francophones had higher rates of mental health-related hospitalizations than Anglophones, it does not appear that a lower level of outpatient psychiatric care was a contributing factor. Indeed, the rates of outpatient and acute psychiatric care in this cohort were low, regardless of language. However, more research is needed to understand the barriers to care that these patients are experiencing. In future studies, it would be important to look at the rate of mental health visits to family physicians in addition to psychiatrist visits to better understand where these patients are receiving care, whether they are receiving adequate care, and what barriers they face.

## Supporting information

S1 Table Description of ICES databases. (DOCX)

S2 Table Detailed study variable definitions. (DOCX)

S3 Table Administrative data codes used to measure comorbid conditions. (DOCX)

S1 Fig Study cohort creation flow diagram. (DOCX)
