# Supplementary material for: Association between preferred language and use of mental health services among home care recipients with schizophrenia spectrum and other psychotic disorders: A retrospective cohort study in Ontario, Canada, 2010 to 2015
Source: PLOS Ment Health. 2024 Jul 22;1(2):e0000013. doi: 10.1371/journal.pmen.0000013 (PMC12798169; doi:10.1371/journal.pmen.0000013)
Supplement: S1 Table — (DOCX) [file pmen.0000013.s001.docx]

S1 Table: Description of ICES databases

| **Database** | **Description** |
| --- | --- |
| Resident Assessment Instrument – Home Care (RAI-HC) | RAI-HC contains data collected from the interRAI Home Care Assessment System that documents client needs, including their health status, physical and cognitive function, living arrangement and supports. |
| Canadian Institute for Health Information Discharge Abstract Database (CIHI-DAD) | CIHI-DAD contains administrative, clinical, and demographic data on all acute care hospital discharges in Ontario. Data include patient demographics, admission and discharge dates, diagnoses (coded using ICD-10 coding scheme), procedures and interventions delivered during inpatient stay (coded via the Canadian Classification of Health Interventions [CCI] coding scheme), intensive care unit admissions, service providers, and discharge disposition. |
| Ontario Mental Health Reporting System (OMHRS) | OMHRS contains data on all patients in adult designated inpatient mental health hospital beds, including general, provincial psychiatric and specialty psychiatric facilities. The Resident Assessment Instrument – Mental Health (RAI-MH) is used to collect the data in OMHRS. Repeated RAI-MH assessments may be conducted during the course of an inpatient mental health stay, including the admission assessment, short stay assessment (when total length of stay is less than 72 hours), change in status assessment, quarterly assessment, and discharge assessment. OMHRS also contains demographic and clinical information on all mental health admissions, including admission and discharge dates, reasons for admission, and diagnoses. |
| National Ambulatory Care Reporting System (NACRS) | NACRS contains administrative, clinical, and demographic data on all emergency department visits in Ontario. Data include patient demographics, visit start and end dates and times, diagnoses (coded using ICD-10 coding scheme), procedures and interventions delivered during visit (coded using CCI coding scheme), physician service providers, and visit disposition. |
| Ontario Drug Benefit Program (ODB) | The ODB contains data on all prescription drugs for those eligible for the ODB program (which includes individuals over 65 years of age, individuals receiving social assistance [Ontario Works, Ontario Disability Support Program], residents of long-term care facilities or homes for special care, home care recipients, Trillium Drug Program and Special Drugs Program recipients). Data includes prescriptions filled (via drug identification numbers), the quantity and number of days supplied, costs, and the date the prescription was filled. |
| Ontario Health Insurance Program Physician Claims (OHIP) | Claims data for all services provided by physicians in Ontario, including in both inpatient and outpatient settings. Physicians paid on non-fee-for-service (FFS) delivery models are required to submit shadow billings for their non-FFS services, with the exception of a few hundred family physicians who work in Community Health Centres in the province. Each billing claim represents a single service provided by the physician, identified by the fee code, as well as a diagnosis code. OHIP also includes claims for laboratory tests conducted outside of hospitals. |
| Registered Persons Database (RPDB) | This is an ICES-derived database that contains demographic information on all individuals who have ever held an Ontario health card, including their date of birth, date of death (where applicable), sex, and OHIP eligibility start and end dates. |
| CENSUS | The Canadian Census contains social data based on a population survey (Census of Population) that include aggregate demographic information such as age, sex, marital status, employment, and income for all persons and housing units within each dissemination area in Canada. Statistics Canada conducts a Census every five years. It takes account of all Canadian citizens (by birth and by naturalization), landed immigrants, and non-permanent residents together with family members living with them. Dissemination areas include between 400-700 persons and the data can be aggregated upward to various geographic levels. |
