# Supplementary material for: Association between preferred language and use of mental health services among home care recipients with schizophrenia spectrum and other psychotic disorders: A retrospective cohort study in Ontario, Canada, 2010 to 2015
Source: PLOS Ment Health. 2024 Jul 22;1(2):e0000013. doi: 10.1371/journal.pmen.0000013 (PMC12798169; doi:10.1371/journal.pmen.0000013)
Supplement: S2 Table — (DOCX) [file pmen.0000013.s002.docx]

S2 Table: Detailed study variable definitions

| **Variable** | **Data holding(s) used for measurement** | **Definition** |
| --- | --- | --- |
| **Exposure** | | |
| Language | RAI-HC | This variable captures an individual’s preferred language for day-to-day communication. It is measured based on observation and interview with the home care recipient and family members, as well as through a review of clinical records. Language is categorized as Anglophone, Francophone, or Allophone, which includes all languages other than English or French. |
| **Outcomes** | | |
| Outpatient psychiatric visits | OHIP | This variable includes all physician visits, based on OHIP physician billing claims, that occurred in an outpatient setting and where the physician specialty was psychiatry. Visit location and specialty is coded in OHIP. We counted a maximum of one visit per day. |
| Mental health-related hospitalizations | CIHI-DAD, OMHRS | Details, including administrative data codes, are included in manuscript. The definition of mental health-related hospitalizations is based on standard approaches for capturing mental health-related encounters using ICES data. |
| Mental health-related ED visits | NACRS | Details, including administrative data codes, are included in manuscript. The definition of mental health-related ED visits is based on standard approaches for capturing mental health-related encounters using ICES data. |
| **Covariates** | | |
| Age | RPDB | Age at the study index date. |
| Sex | RPDB | Male, female |
| Area-level income quintile | CENSUS | The 2016 Canadian census data was used to measure area-level (at the level of census dissemination area) median household income. Area-level income was measured based on individuals’ postal codes at index, which were converted to census areas and linked with income data form the 2016 census. |
| Rurality | RPDB | Individuals’ postal codes were used to assign rurality based on a Statistics Canada definition that indicates the status of enumeration areas based on their population density and proximity to a census metropolitan area/census agglomeration. Rurality as measured based on individuals’ postal codes at their study index date. |
| Count of comorbid conditions | OHIP, CIHI-DAD, ODB | We identified the presence of 16 comorbid conditions used algorithms developed for use in health administrative data. Some of these algorithms (e.g., myocardial infarction, asthma, chronic obstructive pulmonary disease, heart failure, dementia, hypertension, diabetes) have been previously validated. Administrative data diagnosis codes for each condition are provided in S3 Table. We used a count of these conditions as a measure of multimorbidity. |
| Informal helper who lives with individual | RAI-HC | An informal helper lives with a home care recipient if they share the same house, apartment, etc. Response options include yes – an informal helper lives with the recipient, no – an informal helper does not live with the recipient, or the recipient has no such informal helper. |
| Activities of Daily Living (ADL) Self-Performance Hierarchy | RAI-HC | Four items are used to measure the ADL Self-Performance Hierarchy: personal hygiene, toilet use, locomotion in the home, and eating. A composite score is created from the score on these four items to create the ADL Self-Performance Hierarchy, which is scored from 0 to 6, with higher scores indicating greater loss in ADL performance. |
| Cognitive Performance Scale | RAI-HC | Four items are used to measure the Cognitive Performance Scale: short-term memory, cognitive skills for daily decision making, making self understood, and eating. Scores range from 0 to 6, with higher scores indicating more severe cognitive impairment. |
| Changes in Health, End-Stage Disease, Signs and Symptoms (CHESS) scale | RAI-HC | The CHESS scale measures frailty and health instability and was developed to identify individuals at risk of decline. Nine items are used to calculate the CHESS scale: worsening decision making, ADL decline, vomiting, edema, shortness of breath, anticipated less than 6 months to live, unintended weight loss, noticeable decrease in amount of fluids/food consumed, and insufficient fluid consumed. CHESS scores range from 0 to 5, with higher scores indicating a greater risk of decline. |
