# Supplementary material for: Association between preferred language and use of mental health services among home care recipients with schizophrenia spectrum and other psychotic disorders: A retrospective cohort study in Ontario, Canada, 2010 to 2015
Source: PLOS Ment Health. 2024 Jul 22;1(2):e0000013. doi: 10.1371/journal.pmen.0000013 (PMC12798169; doi:10.1371/journal.pmen.0000013)
Supplement: S3 Table — (DOCX) [file pmen.0000013.s003.docx]

S3 Table: Administrative data codes used to measure comorbid conditions

| **Condition** | **ICD-10** | **ICD-9/OHIP fee codes** |
| --- | --- | --- |
| Arthritis - osteoarthritis | M15-M19, M00-M03, M07, M10, M11-M14, M20-M25, M30-M36, M65-M79 | 715, 727, 729, 710, 720, 274, 716, 711, 718, 728, 739 |
| Arthritis – rheumatoid arthritis | M06-M06 | 714 |
| Acute myocardial infarction | I21, I22 | 410 |
| Asthma | J45 | 493 |
| Cancer | C00-C26, C30-C44, C45-C97 | 140-239 |
| Cardiac arrythmia | I48.0, I48.1 | 427 |
| Heart failure | I500, I501, I509 | 428 |
| Chronic obstructive pulmonary disease | J41, J43, J44 | 491, 492, 496 |
| Dementia | F000, F001, F002, F009, F010, F011, F012, F013, F018, F019, F020, F021, F022, F023, F024, F028, F03, F051, F065, F066, F068, F069, F09, G300, G301, G308, G309, G310, G311, R54 | 290, 331, 797 |
| Diabetes | E08 - E13 | E08 - E13 |
| Hypertension | I10, I11, I12, I13, I15 | 401, 402, 403, 404, 405 |
| Osteoporosis | M81, M82 | 733 |
| Renal failure | N17, N18, N19, T82.4, Z49.2, Z99.2 | 403, 404, 584, 585, 586, v451 |
| Stroke | I60-I64 | 430, 431, 432, 434, 436 |
| Coronary artery disease | I20, I22-I25 | 411-414 |
