# Supplementary material for: Association between preferred language and use of mental health services among home care recipients with schizophrenia spectrum and other psychotic disorders: A retrospective cohort study in Ontario, Canada, 2010 to 2015
Source: PLOS Ment Health. 2024 Jul 22;1(2):e0000013. doi: 10.1371/journal.pmen.0000013 (PMC12798169; doi:10.1371/journal.pmen.0000013)
Supplement: S1 Fig — (DOCX) [file pmen.0000013.s004.docx]

S1 Figure: Study cohort creation flow diagram
